# Pretreatment of therapeutic cells with poly(ADP-ribose) polymerase inhibitor enhances their efficacy in an *in vitro* model of cell-based therapy in myocardial infarct

**DOI:** 10.3892/ijmm.2012.1186

**Published:** 2012-11-16

**Authors:** MÓNIKA SZEPES, ZSÓFIA JANICSEK, ZSOLT BENKŐ, ATTILA CSELENYÁK, LEVENTE KISS

**Affiliations:** Institute of Human Physiology and Clinical Experimental Research, Semmelweis University, Budapest, Hungary

**Keywords:** cell-based therapy, ischemia-reperfusion, poly(ADP-ribose) polymerase, pretreatment

## Abstract

The potential of cell-based therapies in diseases involving ischemia-reperfusion is greatly hampered by the excessive loss of administered cells in the harsh and oxidative environment where these cells are supposed to act. Therefore, we investigated if inhibition of poly(ADP-ribose) polymerase (PARP) in the therapeutically added cells would lead to their increased viability and, subsequently, to an enhanced effect in an *in vitro* simulated ischemia-reperfusion (I-R) setting. Ischemic conditions were simulated by oxygen and glucose deprivation for 160 min using H9c2 rat cardiomyoblast cells. After 30 min of reperfusion, these cells received 4 types of treatments: no added cells (I-R model), fluorescently labeled (Vybrant DiD) therapeutic H9c2 cells with vehicle (H9c2) or PARP inhibitor (10 μM or 100 μM PJ34) pretreatment. We assessed viability (live, apoptotic and necrotic) of both ‘postischemic’ and therapeutic cells with flow cytometric analysis using calcein-AM/ethidium homodimer-2 fluorescent staining after 24 h of co-culture. Further measurements on necrosis and metabolic activity were performed using lactate dehydrogenase (LDH) release and resazurin based assays. The percentage of surviving therapeutic cells increased significantly with PARP inhibition (untreated, 52.02±5.01%; 10 μM PJ34, 63.38±4.50%; 100 μM PJ34, 64.99±3.47%). The percentage of necrotic cells decreased in a similar manner (untreated, 37.23±4.40%; 10 μM PJ34, 26.83±3.49%; 100 μM PJ34, 24.96±2.43%). Notably, the survival of the cells that suffered I-R injury was also significantly higher when treated with PARP-inhibited therapeutic cells (I-R model, 36.44±5.05%; H9c2, 42.81±5.11%; 10 μM PJ34, 52.07±5.80%; 100 μM PJ34, 54.95±5.55%), while necrosis was inhibited (I-R model, 43.64±4.00%; H9c2, 37.29±4.55%; 10 μM PJ34, 30.18±4.60%; 100 μM PJ34, 25.52±3.47%). In subsequent experiments, PARP inhibition decreased LDH-release of the observed combined cell population and enhanced the metabolic activity. Thus, our results suggest that pretreating the therapeutically added cells with a PARP inhibitor could be beneficial in the setting of cell-based therapies.

## Introduction

Regenerating the heart through cell transplantation is a promising novel approach in the therapy of myocardial infarct and various investigations have provided evidence that this approach has indeed the potential to improve the functionality of the injured heart (1,2). However, a meta-analysis with nearly a thousand patients concluded that bone marrow derived cell transplantation resulted in only a modest 3.66% improvement of the left ventricular ejection fraction in patients with ischemic heart disease (3–5), which falls well below the expectations towards this new therapeutic approach. These disappointing results could be the consequence of the unclear underlying mechanism of action of the therapeutic cells which may involve various mechanisms such as paracrine and direct cell-to-cell effects (6–10). Understanding the mechanisms of action could lead to better optimalization of the used protocols. On the other hand, most of the therapeutically added cells die in the noxious and aggressive environment of ‘postischemic’ myocardium (11–13). While some experimental evidence suggest that the effect is mostly due to apoptotic cells which secrete factors that could protect cells after myocardial infarct (14), it is reasonable to hypothesize that if more cells survived after grafting, then their actions, either paracrine or cell-to-cell, could be more efficient and, thus, the treatments could be more effective. Evidence supporting this concept has already been validated using various pretreatment methods, such as ‘priming’ with growth factors or modifying with Akt (15–18). A further possibility to enhance survival could be to prepare cells for the oxidative stress present in the reperfused cardiac tissue. Oxidative stress induced pathways play a major role in the development of ‘postischemic’ injuries in the heart (19–21) and these involve poly(ADP-ribose) polymerase (PARP) activation (22,23). PARP is an energy-consuming enzyme that functions primarily as a DNA damage sensor in the nucleus and catalyzes the cleavage of NAD^+^ into nicotinamide and ADP-ribose, then transfers ADP ribose units to nuclear proteins such as histons and transcription factors. As a result of this process, the intracellular NAD^+^ and ATP levels remarkably decrease, resulting in cell dysfunction and cell death (24). Recent studies have also implicated the importance of mitochondrial dysfunction and mitochondrial cell death factors, including apoptosis-inducing factors in the process of oxidant-induced cell death and the potential role of PARP in regulating these factors in various cell types including myocardial cells (25–28).

Previous studies have demonstrated the direct protective effect of PARP inhibition of cells or tissues undergoing ischemia-reperfusion (I-R) injury (23,29–31). Our aim was to assess the potential of PARP inhibitor pretreatment in a cell-based therapy setting where the added therapeutic cells received the pretreatment. Accordingly, we used a reductionist *in vitro* model of cell-based therapy in myocardial infarct where the therapeutically added cells were pretreated with PARP inhibitor and we investigated if improved survival of the therapeutic cells could enhance the viability of cells undergoing simulated I-R injury.

## Materials and methods

### Cell culture

H9c2 rat cardiomyoblasts were purchased from ATCC (Wesel, Germany). Cells were cultured in high glucose (4.5 g/l) DMEM containing 10% fetal bovine serum, 4 mM L-glutamine, 100 U/ml penicillin and 100 μg/ml streptomycin at 37°C in a humidified atmosphere of 5% CO_2_. Cell culture media were changed every 2–3 days and cells were sub-cultured once they reached 70–80% confluence. Cells between passages 7 and 13 were used in the experiments.

### Simulated ischemia-reperfusion model

Myocardial I-R was simulated *in vitro* on H9c2 rat cardiomyoblast cell cultures based on the method of Cselenyák *et al*(9) with modifications. Briefly, to mimic the ischemic conditions, cells were incubated in glucose-free DMEM in an atmosphere of 0.5% O_2_ and 99.5% N_2_ for 160 min on the stage of a confocal microscope (PeCon Incubation System, Erbach-Bach, Germany). Glucose was replaced with fresh high glucose DMEM and the cells were placed in standard cell culture conditions (37°C, 5% CO_2_) until further experimental actions.

### Malondialdehyde measurement

Malondialdehyde (MDA) formation was used to quantify the lipid peroxidation in our simulated I-R model and was measured as thiobarbituric acid reactive material. According to the detection limit of the assay protocol 1,000,000 cells were used. Five hours after the start of simulated reperfusion 50 μl of the cell culture supernatant was added to a reaction mixture consisting of 50 μl of 8.1% sodium dodecyl sulfate, 375 μl of 20% acetic acid (pH 3.5), and 150 μl of distilled water. The mixture was completed with 375 μl of freshly prepared, boiling hot thiobarbituric acid (0.8%) and incubated at 95°C for 1 h. After cooling to room temperature, 200 μl supernatant was transferred into 96-well microplates and the absorbance was measured at 532 nm (32). Malonaldehyde bis(dimethyl acetal), thiobarbituric acid and sodium dodecyl sulfate were purchased from Sigma-Aldrich (St. Louis, MO, USA).

### In vitro model of cell based therapy

**Figure 1:**
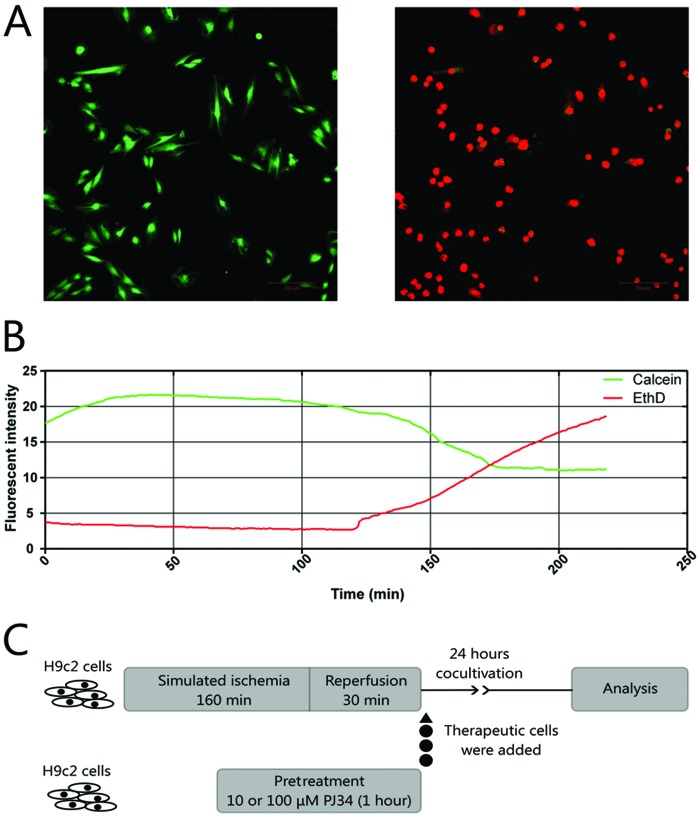
Experimental setup. (A) Confocal microscopy images taken before and after oxygen glucose deprivation (green, calcein-AM; red, EthD). (B) Changes in fluorescent intensity of live/dead staining during simulated ischemia. (C) Experimental protocol.

Based on our previous experiments (33), we used H9c2 cells as therapeutic cells which were added to the damaged cells 30 min after the start of simulated reperfusion and set a simulated ischemia so severe that the untreated therapeutic cells were not able to improve survival. The optimal time of simulated ischemia was set using time lapse video microscopy to follow the changes in the fluorescent intensity of calcein-AM/ethidium-homodimer-2 (EthD) viability staining (Fig. 1A). After ~2 h, rapid decrease occurred in the calcein-AM intensity and at the same time the intensity of the dead cell indicator EthD increased (Fig. 1B). For the experiments we chose an ischemia length of 160 min. The used therapeutic cells were divided into 3 experimental groups according to their pretreatment protocol lasting 1 h: 1) control group treated with saline vehicle; 2) 10 μM PJ34 (PARP inhibitor; Inotek Pharmaceuticals Corp., Beverly, MA, USA); 3) 100 μM PJ34. After the pretreatment, the H9c2 cells were washed twice with PBS to ensure no PARP inhibitor remained, trypsinized, collected by centrifugation and 20,000 cells were given the appropriate wells of the 12-well plate containing 30,000 ‘postischemic’ cells that underwent simulated I-R. Cells were co-cultivated for 24 h before further flow cytometric, lactate dehydrogenase (LDH) release and metabolic activity measurements (Fig. 1C).

### Lactate dehydrogenase measurements

We used measurements of LDH release for three reasons: i) to evaluate the I-R model; ii) to describe the cytotoxicity and efficacy of PJ34 on our H9c2 cells; iii) to compare the cumulated necrosis of cells in the experimental groups of our cell-therapy model. In the evaluation of the model, 100,000 H9c2 cells were used and measured at 24 h after the start of reperfusion. For the evaluation of the PJ34 PARP inhibitor, we measured an untreated group and groups pretreated with 10 or 100 μM PJ34. After incubation with vehicle or PJ34 for 1 h, 10,000 cells were placed in a well and each group was either treated with vehicle to evaluate the cytotoxicity of the inhibitor or given 400 μM H_2_O_2_ for 2 h to check the inhibitor’s efficacy. Measurements were standardized using untreated cells and cells were lysed using 1% Triton X-100 or cell lysis solution to obtain the background and the total LDH content. In the third case, the LDH level was estimated in cell culture supernatant 24 h after reperfusion according to the manufacturer’s instructions (LDH Cytotoxicity kit I and II; PromoCell, Heidelberg, Germany) and differences compared to the vehicle treated group (H9c2) were given. The absorbance was taken at 450 and 650 nm and the percentage of cytotoxicity was calculated.

### Flow cytometric measurements

**Figure 2:**
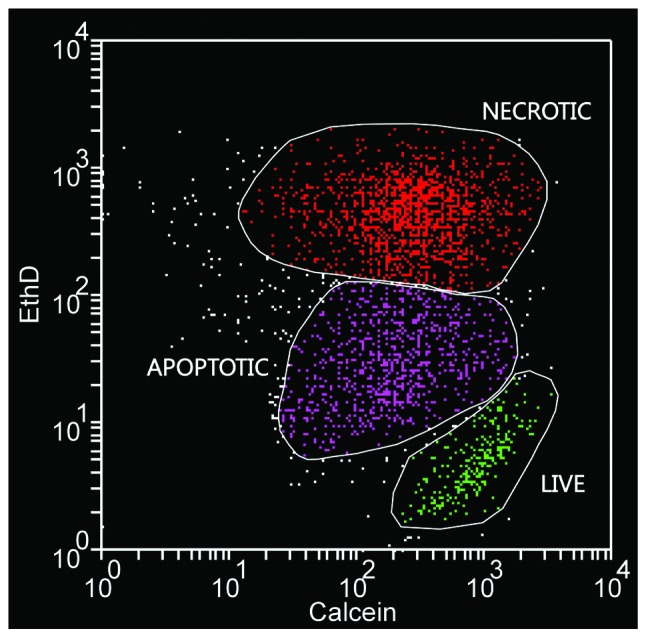
Representative image of the three groups found during the analysis of cell survival (green, calcein-AM positive living cells; red, ethidium-homodimer-2 positive necrotic cells; violet, double positive apoptotic cells).

Flow cytometric analysis was performed 24 h after reperfusion (FACSCalibur™; Becton-Dickinson, Franklin Lakes, NJ, USA). Cells were harvested by trypsinization and resuspended in 500 μl PBS containing 250 nM calcein-AM (ex/em, 494/517 nm; Invitrogen, Carlsbad, CA, USA) and 350 nM ethidium-homodimer-2 (ex/em, 536/624 nm; Invitrogen). Using flow cytometry we could distinguish the therapeutically given cells from the ‘postischemic’ cells on the basis of their Vybrant DiD labeling (ex/em, 633/665 nm; Molecular Probes, USA) and these cells were gated in or out as appropriate for further analysis. Evaluation of cell death was performed according to Palma *et al*(34). Briefly, maximally ethidium homodimer-2 positive and slightly calcein-AM negative cells were considered necrotic, ethidium homodimer-2 negative but calcein-AM positive cells were counted as live and a distinct group of cells with intermediate staining characteristics was considered as apoptotic (Fig. 2).

### Metabolic activity

For the evaluation of the metabolic activity in the experimental groups we used PrestoBlue™ cell viability reagent (Invitrogen). PrestoBlue is a resazurin-based reagent that functions as a cell viability indicator by using the reducing power of living cells. Cardiomyoblasts were prepared on a 12-well plate and therapeutic cells were added after simulated I-R as previously described. After 24 h of co-cultivation, the culture media was replaced by phenol red free high glucose DMEM containing 10% PrestoBlue cell viability reagent. Following a further 24 h of incubation, 200 μl cell culture supernatant was taken from all samples and added into the wells of a 96-well plate. The absorbance was measured at 570 and 600 nm, according to the manufacturer’s description, and the 570 nm values were normalized to the 600 nm values.

### Statistical analysis

The flow cytometric measurements were evaluated using Weasel software (WEHI, Australia). Statistical analysis of data was carried out using one-way analysis of variance with the Newman-Keuls multiple comparison post hoc test or the Student’s t-test as appropriate. All data are expressed as the means ± SEM. A P-value of <0.05 was considered to indicate statistically significant differences.

## Results

### Simulated ischemia-reperfusion protocol

**Figure 3:**
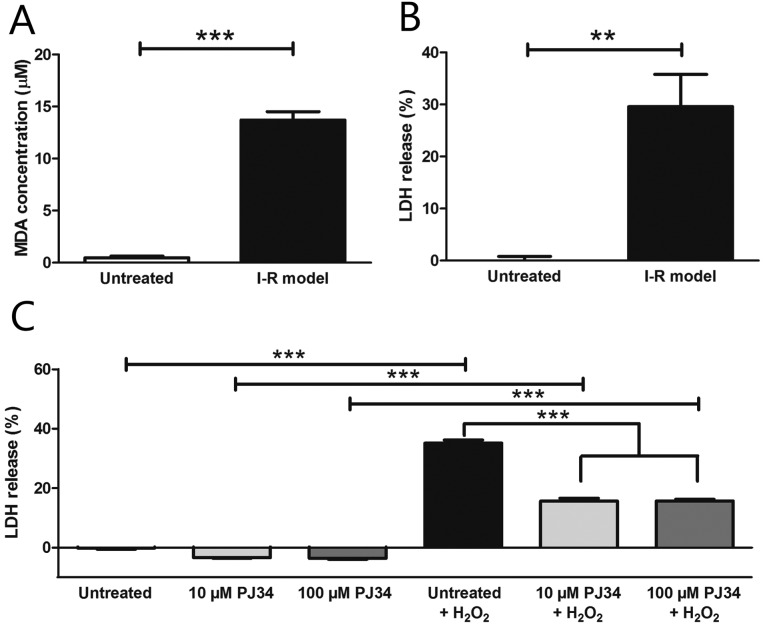
Validation of the experimental model. (A) Concentration of the lipid peroxidation product MDA in the supernatant (^***^P<0.001 mean ± SEM, n=6). (B) Ratio of necrotized cells on the basis of LDH assays (^**^P<0.01 mean ± SEM, n=12); (C) PJ34 cytotoxicity and inhibitor efficacy (^***^P<0.001 mean ± SEM, n=6).

The level of the oxidative stress based on MDA assay significantly increased in our I-R model (13.70±0.81 μM) compared to untreated cells (0.47±0.18 μM) (Fig. 3A). Oxygen glucose deprivation led to a significant increase in the LDH levels in the supernatant compared to the control conditions (untreated, 0.00±0.81%; I-R model, 29.58±6.21%) (Fig. 3B). The PARP inhibitor PJ34 did not exert any cytotoxic effect in the used concentrations in our H9c2 cells and significantly protected these cells against H_2_O_2_ induced injury in both concentrations (untreated + H_2_O_2_, 35.14±1.01%; 10 μM PJ34 + H_2_O_2_, 15.65±0.95%; 100 μM PJ34 + H_2_O_2_, 15.69±0.54%) (Fig. 3C).

### Flow cytometric measurements

**Figure 4:**
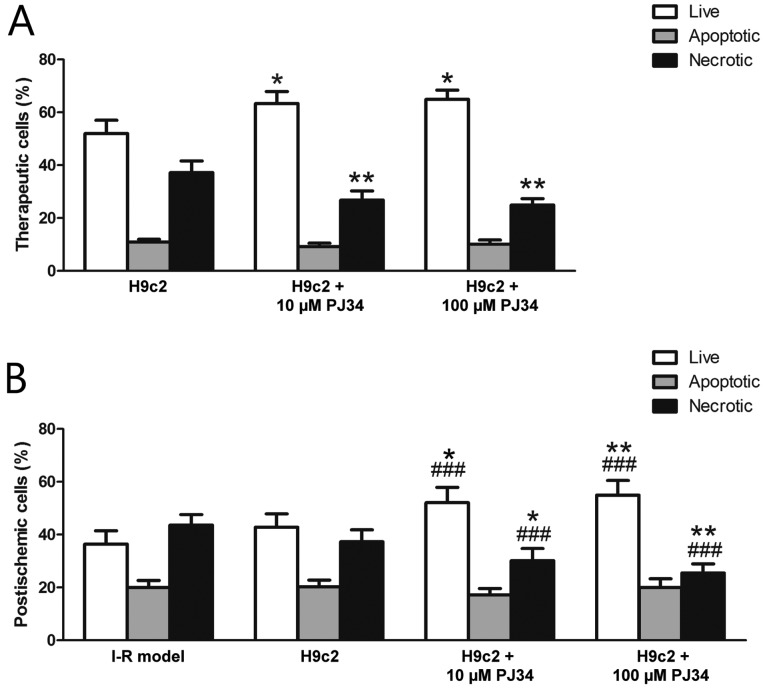
Results of flow cytometric live/dead analysis. (A) The ratio of living, apoptotic and necrotic therapeutically added cells (^*^P<0.05 vs. H9c2, ^**^P<0.01 vs. H9c2, mean ± SEM, n=18). (B) The ratio of living, apoptotic and necrotic cells in the ‘postischemic’ groups (^*^P<0.05 vs. H9c2, ^**^P<0.01 vs. H9c2, ^###^P<0.001 vs. I-R model, mean ± SEM, n=18).

Flow cytometric analysis of live, apoptotic and necrotic populations of the therapeutically added cells showed significantly increased survival with PARP inhibition (H9c2, 52.02±5.01%; H9c2 + 10 μM PJ34, 63.38±4.50%; H9c2 + 100 μM PJ34, 64.99±3.47%), while the ratio of necrotic cells decreased when PJ34 pretreatment was used (H9c2, 37.23±4.40%; H9c2 + 10 μM PJ34, 26.83±3.49%; H9c2 + 100 μM PJ34, 24.96±2.43%). No differences were found among the ratios of apoptotic cells (H9c2, 10.87±1.12%; H9c2 + 10 μM PJ34, 9.22±1.28%; H9c2 + 100 μM PJ34, 10.18±1.55%) (Fig. 4A).

There was no significant difference between the I-R model and the H9c2 treated group regarding live (I-R model, 36.44±5.05%; H9c2, 42.81±5.11%) and necrotic (I-R model, 43.64±4.00%; H9c2, 37.29±4.55%) ratios. This was expected as the experimental damage was set to acquire such data. However, and importantly, the survival of the ‘postischemic’ cells was higher when treated with PARP-inhibited therapeutic cells (H9c2 + 10 μM PJ34, 52.07±5.80%; H9c2 + 100 μM PJ34, 54.95±5.55%) and the ratio of necrotic cells also decreased (H9c2 + 10 μM PJ34, 30.18±4.60%; H9c2 + 100 μM PJ34, 25.52±3.47%). The percentages of apoptotic cells did not show a statistically significant difference among the groups (I-R model, 19.94±2.75%; H9c2, 20.23±2.62%; H9c2 + 10 μM PJ34, 17.20±2.42%; H9c2 + 100 μM PJ34, 20.05±3.23%) (Fig. 4B).

### Lactate dehydrogenase cytotoxicity assay

**Figure 5:**
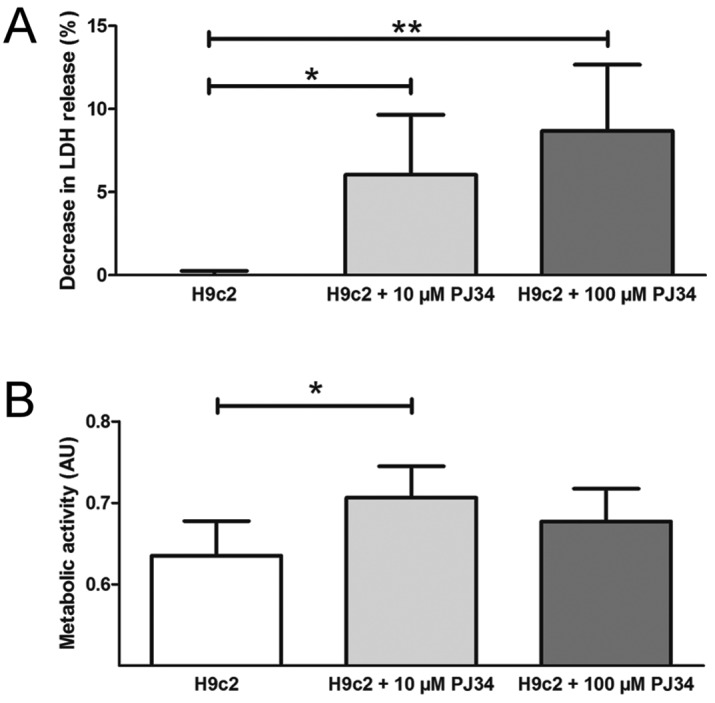
Cytotoxicity and viability measurements. (A) Decrease in LDH release on an inverted scale (^*^P<0.05, ^**^P<0.01 mean ± SEM, n=7). (B) Metabolic activity measured by PrestoBlue reagent (^*^P<0.05 mean ± SEM, n=8).

Cellular necrosis expressed by LDH release decreased significantly when the therapeutic cells were pretreated with the PARP inhibitor. Using 10 μM and 100 μM PJ34 the LDH release decreased with 6.04±3.61% and 8.68±3.98%, respectively (Fig. 5A).

### Metabolic activity

PARP inhibition significantly enhanced the overall metabolic activity of the cells when therapeutic cells were treated with 10 μM PJ34 compared to untreated H9c2 (H9c2, 0.64±0.04%; 10 μM PJ34, 0.71±0.04%; arbitrary units). There was only a nonsignificant trend of such increase when a 100 μM concentration PJ34-treated cardiomyoblasts were used (0.68±0.04%) (Fig. 5B).

## Discussion

Herein we report that the pretreatment of therapeutic cells improves their survival and subsequently increases the viability of the damaged cardiomyoblasts in an *in vitro* reductionist model of cell-based therapy in myocardial infarct. First, we evaluated our experimental *in vitro* model for oxidative stress, necrotic properties and we checked the cytotoxicity and efficacy of the used PARP inhibitor. We found that following oxygen and glucose deprivation, the MDA levels are increased, as it can be observed in *in vivo* ischemic conditions. According to our measurements on cell membrane integrity based on LDH level, the simulated ischemia is followed by significant membrane damage. Thus the applied model properly simulates the I-R injury. The earlier data on the PARP inhibitor were confirmed regarding its cytotoxicity and efficacy in the used concentrations (29). This measurement also indicates that our *in vitro* simulated ischemia model caused damage similar to a 400 μM H_2_O_2_ treatment for 2 h. The timing of the cell addition was partly chosen based on the literature that suggests non-immediate delivery of cells (35) and partly on our own pilot experiments that also suggested better efficacy if cells were given 30 min after the start of reperfusion (data not shown).

Using flow cytometry we showed that PARP inhibition of the therapeutic cells could improve the viability of the ‘postischemic’ cells. The mechanism of this beneficial effect seems to be connected to the increased ratio of surviving therapeutic cells. It appears, therefore, that the therapeutic cells with PJ34 pretreatment could help damaged cells to survive. Untreated therapeutic cells had no significant effect on this cell population. Imaging a real myocardial infarct it may mean that areas with bigger oxidative damage could also be saved with such pretreated therapeutic cells. Regarding the exact mechanism of the therapeutic cells we assume based on our earlier observations (9,33) and on the results of others (8,36), that this beneficial effect could be related partly to cell-to-cell connections and partly to paracrine factors released from the therapeutic cells.

If we consider the possible mechanisms related to the improved survival of therapeutic cells we must remember that reactive oxygen species are believed to play a key role in the myocardial I-R injury and myocardial cell death in I-R is mediated mainly by necrosis and the mechanism is based on the oxidative stress-induced activation of PARP (37). It is important to realize at this point that the PARP inhibitor treatment occurred before adding the therapeutic cells to the damaged ones, therefore these latter cells did not receive any pretreatment. The protective effect of PJ34 is extended beyond the end of the treatment and pharmacokinetic data indicate that the prolonged effect of PJ34 is not related to the continued presence of the inhibitor, but it may be related to the permanent interruption of positive-feedback cycles of injury. Earlier studies have demonstrated that PARP inhibitors block positive-feedback cycles of adhesion-receptor expression and mononuclear cell infiltration, as well as intracellular oxidant generation (24). A possible concern could be the potential genotoxic nature of PARP inhibitors as these block the DNA-repair mechanisms. Results from earlier studies disprove this as one study showed that the inhibition of poly(ADP-ribosyl)ation did not particularly modify genotoxicity (38) while another, using bacterial reverse mutation test, *in vitro* chromosomal aberration test and bone marrow micronucleus test concluded that PARP inhibition did not possess genotoxic activity (39).

In our subsequent experiments, the effect of PARP inhibition pretreatment was examined using LDH and PrestoBlue measurements. These two methods reflect the necrosis and metabolic activity of both cell populations after 24 h, respectively. LDH values significantly decreased with both applied concentrations of PJ34. Pretreatment with 10 μM PJ34 significantly increased the metabolic activity in the cell culture compared to untreated H9c2, but in the case of 100 μM PJ34 we saw only a non-significant trend towards the increased value. Blocking PARP activation as much as it is possible should lead, at least in theory, to better protection. The higher concentration in our experiments, however, could not improve the beneficial effect further or in the case of PrestoBlue it was only a non-significant increase. Thus, we suspect that 10 μM PJ34 fully blocks the PARP enzyme in H9c2 cells after 1 h. The results that the higher concentration of PJ34 did not significantly improve the metabolic activity of ‘postischemic’ cells may reflect that higher concentration of PARP inhibitors could cause metabolic suppression, but this hypothesis warrants further investigations. As a limitation to the interpretation of these measurements, it must be realized that the combined LDH levels and metabolic activities of the two cell populations were measured. However, as these factors are frequently used to reflect the viability of cell groups we believe that the improved combined values provide valuable data to our study and further strengthen our findings.

There are some other limitations to our study as well. First, the demonstrated protocol is an *in vitro* approach to the much more complex issue of stem cell therapy in myocardial infarct, with all the advantages and disadvantages of such a model. Obviously, it cannot reflect the complex (e.g. immunological) events taking place during and after myocardial infarct but the parameters (time of the ischemia and reperfusion, the temperature and the O_2_ concentration) can easily be controlled and the effects of the added cells on the ‘postischemic’ cells can be quantified. The 24 h co-cultivation time period was chosen based on preliminary experiments as this time was found to suffice for the attachment of the newly added cells but was not enough for proliferation. Second, we used H9c2 cells as a model for cardiac muscle and although the cell line is frequently used in similar experiments they resemble skeletal muscle cells just as much as cardiac muscle (40,41). Thus, conclusions drawn from results obtained from them should be considered cautiously. Another concern could be the fact that we used H9c2 cells as therapeutic cells. Our earlier experimental evidence showed that healthy H9c2 cells could save their oxidatively damaged neighbors from delayed death (33). Furthermore, since we obtained similar effectiveness with stem cells and H9c2 cells in that model, it reflects that multipotency of the grafted cells is not required for this type of rescue effect, widening the possible sources for cell therapy.

Our data are in line with other observations on pretreatments in the literature. Yao *et al*(15) transplanted mesenchymal stem cells pretreated with lipopolysaccharide into an *in vivo* rat model. They found better vascularisation, lower fibrosis in the myocardium, and the viability of the therapeutic cells improved. Hahn *et al*(16) used a growth factor combination for pretreatment and checked the survival of the cells after 0.5% hypoxia for 30 h. They found a 20% increase in trypan blue exclusion. This effect is quantitatively similar to the effect of our PJ34 inhibition achieved in the H_2_O_2_ protocol. Mouse BMSCs were pretreated with H_2_O_2_ for 30 min in the Kubo *et al*(18) model. The production of vascular endothelial growth factor was increased and the differentiation towards endothelial cells was promoted. Mangi *et al*(17) pretreated therapeutic cells with retroviral manipulation for overexpressing Akt1 which restored the myocardium more effectively than the control cells. Of note, however, we believe that our study presents the first quantitative viability data on the effect of therapeutic cell pretreatment on the ‘postischemic’ cells.

In conclusion, addition of PARP-inhibited cells to severely injured cardiomyoblasts can improve survival and decrease cellular necrosis in an *in vitro* cell therapy model for I-R injury. This approach, if confirmed in experiments using human derived cells and in *in vivo* studies, could lead to a better efficacy of cell-based therapies with a relatively simple and economical pretreatment step.

## References

[1] Dill T, Schachinger V, Rolf A (2009). Intracoronary administration of bone marrow-derived progenitor cells improves left ventricular function in patients at risk for adverse remodeling after acute ST-segment elevation myocardial infarction: results of the Reinfusion of Enriched Progenitor cells And Infarct Remodeling in Acute Myocardial Infarction study (REPAIR-AMI) cardiac magnetic resonance imaging substudy. Am Heart J.

[2] Medicetty S, Wiktor D, Lehman N (2011). Percutaneous adventitial delivery of allogeneic bone marrow derived stem cells via infarct related artery improves long-term ventricular function in acute myocardial infarction. Cell Transplant.

[3] Paul D, Samuel SM, Maulik N (2009). Mesenchymal stem cell: present challenges and prospective cellular cardiomyoplasty approaches for myocardial regeneration. Antioxid Redox Signal.

[4] Penn MS, Dong F, Klein S, Mayorga ME (2011). Stem cells for myocardial regeneration. Clin Pharmacol Ther.

[5] Abdel-Latif A, Bolli R, Tleyjeh IM (2007). Adult bone marrow-derived cells for cardiac repair: a systematic review and meta-analysis. Arch Intern Med.

[6] Dayan V, Yannarelli G, Billia F (2011). Mesenchymal stromal cells mediate a switch to alternatively activated monocytes/macrophages after acute myocardial infarction. Basic Res Cardiol.

[7] Ramos GA, Hare JM (2007). Cardiac cell-based therapy: cell types and mechanisms of actions. Cell Transplant.

[8] Gnecchi M, Zhang Z, Ni A, Dzau VJ (2008). Paracrine mechanisms in adult stem cell signaling and therapy. Circ Res.

[9] Cselenyák A, Pankotai E, Horváth E, Kiss L, Lacza Z (2010). Mesenchymal stem cells rescue cardiomyoblasts from cell death in an in vitro ischemia model via direct cell-to-cell connections. BMC Cell Biol.

[10] Plotnikov EY, Khryapenkova TG, Vasileva AK (2008). Cell-to-cell cross-talk between mesenchymal stem cells and cardiomyocytes in co-culture. J Cell Mol Med.

[11] Wu KH, Mo XM, Han ZC, Zhou B (2011). Stem cell engraftment and survival in the ischemic heart. Ann Thorac Surg.

[12] Singla DK, Singla RD, Lamm S, Glass C (2011). TGF-beta2 treatment enhances cytoprotective factors released from embryonic stem cells and inhibits apoptosis in infarcted myocardium. Am J Physiol Heart Circ Physiol.

[13] Singla DK, McDonald DE (2007). Factors released from embryonic stem cells inhibit apoptosis of H9c2 cells. Am J Physiol Heart Circ Physiol.

[14] Lichtenauer M, Mildner M, Hoetzenecker K (2011). Secretome of apoptotic peripheral blood cells (APOSEC) confers cytoprotection to cardiomyocytes and inhibits tissue remodelling after acute myocardial infarction: a preclinical study. Basic Res Cardiol.

[15] Yao Y, Zhang F, Wang L (2009). Lipopolysaccharide preconditioning enhances the efficacy of mesenchymal stem cells transplantation in a rat model of acute myocardial infarction. J Biomed Sci.

[16] Hahn JY, Cho HJ, Kang HJ (2008). Pre-treatment of mesenchymal stem cells with a combination of growth factors enhances gap junction formation, cytoprotective effect on cardiomyocytes, and therapeutic efficacy for myocardial infarction. J Am Coll Cardiol.

[17] Mangi AA, Noiseux N, Kong D (2003). Mesenchymal stem cells modified with Akt prevent remodeling and restore performance of infarcted hearts. Nat Med.

[18] Kubo M, Li TS, Suzuki R, Ohshima M, Qin SL, Hamano K (2007). Short-term pretreatment with low-dose hydrogen peroxide enhances the efficacy of bone marrow cells for therapeutic angiogenesis. Am J Physiol Heart Circ Physiol.

[19] Hausenloy DJ (2009). Signalling pathways in ischaemic postconditioning. Thromb Haemost.

[20] Yellon DM, Hausenloy DJ (2007). Myocardial reperfusion injury. N Engl J Med.

[21] Hori M, Nishida K (2009). Oxidative stress and left ventricular remodelling after myocardial infarction. Cardiovasc Res.

[22] Pacher P, Szabo C (2007). Role of poly(ADP-ribose) polymerase 1 (PARP-1) in cardiovascular diseases: the therapeutic potential of PARP inhibitors. Cardiovasc Drug Rev.

[23] Sodhi RK, Singh N, Jaggi AS (2010). Poly(ADP-ribose) polymerase-1 (PARP-1) and its therapeutic implications. Vascul Pharmacol.

[24] Virag L, Szabo C (2002). The therapeutic potential of poly(ADP-ribose) polymerase inhibitors. Pharmacol Rev.

[25] Ullrich O, Diestel A, Eyupoglu IY, Nitsch R (2001). Regulation of microglial expression of integrins by poly(ADP-ribose) polymerase-1. Nat Cell Biol.

[26] Jagtap P, Szabo C (2005). Poly(ADP-ribose) polymerase and the therapeutic effects of its inhibitors. Nat Rev Drug Discov.

[27] Wang Y, Kim NS, Haince JF (2011). Poly(ADP-ribose) (PAR) binding to apoptosis-inducing factor is critical for PAR polymerase-1-dependent cell death (parthanatos). Sci Signal.

[28] Koh DW, Dawson TM, Dawson VL (2005). Mediation of cell death by poly(ADP-ribose) polymerase-1. Pharmacol Res.

[29] Fiorillo C, Ponziani V, Giannini L (2006). Protective effects of the PARP-1 inhibitor PJ34 in hypoxic-reoxygenated cardiomyoblasts. Cell Mol Life Sci.

[30] Oh KS, Lee S, Yi KY, Seo HW, Koo HN, Lee BH (2009). A novel and orally active poly(ADP-ribose) polymerase inhibitor, KR-33889 [2-[methoxycarbonyl(4-methoxyphenyl) methylsulfanyl]-1H-benzimidazole-4-carboxylic acid amide], attenuates injury in in vitro model of cell death and in vivo model of cardiac ischemia. J Pharmacol Exp Ther.

[31] Song ZF, Ji XP, Li XX, Wang SJ, Wang SH, Zhang Y (2008). Inhibition of the activity of poly(ADP-ribose) polymerase reduces heart ischaemia/reperfusion injury via suppressing JNK-mediated AIF translocation. J Cell Mol Med.

[32] Liaudet L, Mabley JG, Soriano FG (2001). Inosine reduces systemic inflammation and improves survival in septic shock induced by cecal ligation and puncture. Am J Respir Crit Care Med.

[33] Pankotai E, Cselenyak A, Ratosi O, Lorincz J, Kiss L, Lacza Z (2012). The role of mitochondria in direct cell-to-cell connection dependent rescue of postischemic cardiomyoblasts. Mitochondrion.

[34] Palma PF, Baggio GL, Spada C, Silva RD, Ferreira SI, Treitinger A (2008). Evaluation of annexin V and Calcein-AM as markers of mononuclear cell apoptosis during human immunodeficiency virus infection. Braz J Infect Dis.

[35] Dimmeler S, Burchfield J, Zeiher AM (2008). Cell-based therapy of myocardial infarction. Arterioscler Thromb Vasc Biol.

[36] Uemura R, Xu M, Ahmad N, Ashraf M (2006). Bone marrow stem cells prevent left ventricular remodeling of ischemic heart through paracrine signaling. Circ Res.

[37] Burkle A (2001). Physiology and pathophysiology of poly(ADP-ribosyl)ation. Bioessays.

[38] Oliveira NG, Castro M, Rodrigues AS (2005). Effect of poly(ADP-ribosyl)ation inhibitors on the genotoxic effects of the boron neutron capture reaction. Mutat Res.

[39] Vinod KR, Chandra S, Sharma SK (2010). Evaluation of 5-aminoisoquinoline (5-AIQ), a novel PARP-1 inhibitor for genotoxicity potential in vitro and in vivo. Toxicol Mech Methods.

[40] Kimes BW, Brandt BL (1976). Properties of a clonal muscle cell line from rat heart. Exper Cell Res.

[41] Sardao VA, Oliveira PJ, Holy J, Oliveira CR, Wallace KB (2007). Vital imaging of H9c2 myoblasts exposed to tert-butylhydroperoxide - characterization of morphological features of cell death. BMC Cell Biol.

